# Glucose-6 Phosphate, a Central Hub for Liver Carbohydrate Metabolism

**DOI:** 10.3390/metabo9120282

**Published:** 2019-11-20

**Authors:** Fabienne Rajas, Amandine Gautier-Stein, Gilles Mithieux

**Affiliations:** 1 Institut National de la Santé et de la Recherche Médicale, U1213, F-69008 Lyon, France; amandine.gautier-stein@univ-lyon1.fr (A.G.-S.); gilles.mithieux@univ-lyon1.fr (G.M.); 2 Université de Lyon, F-69008 Lyon, France; 3 Université Lyon 1, F-69622 Villeurbanne, France

**Keywords:** de novo lipogenesis, carbohydrate response element-binding protein, ChREBP, diabetes, glucose production, glycogen, glycolysis, glycogen storage disease type I, hexosamine, nonalcoholic fatty liver disease, NAFLD, pentose phosphate pathway, steatosis

## Abstract

Cells efficiently adjust their metabolism according to the abundance of nutrients and energy. The ability to switch cellular metabolism between anabolic and catabolic processes is critical for cell growth. Glucose-6 phosphate is the first intermediate of glucose metabolism and plays a central role in the energy metabolism of the liver. It acts as a hub to metabolically connect glycolysis, the pentose phosphate pathway, glycogen synthesis, de novo lipogenesis, and the hexosamine pathway. In this review, we describe the metabolic fate of glucose-6 phosphate in a healthy liver and the metabolic reprogramming occurring in two pathologies characterized by a deregulation of glucose homeostasis, namely type 2 diabetes, which is characterized by fasting hyperglycemia; and glycogen storage disease type I, where patients develop severe hypoglycemia during short fasting periods. In these two conditions, dysfunction of glucose metabolism results in non-alcoholic fatty liver disease, which may possibly lead to the development of hepatic tumors. Moreover, we also emphasize the role of the transcription factor carbohydrate response element-binding protein (ChREBP), known to link glucose and lipid metabolisms. In this regard, comparing these two metabolic diseases is a fruitful approach to better understand the key role of glucose-6 phosphate in liver metabolism in health and disease.

## 1. Introduction

The liver plays a crucial role in the maintenance of glucose homeostasis by extracting glucose from the blood and then storing it after a meal, and also by producing glucose in post-absorptive state. When its concentration increases in the bloodstream, glucose enters the hepatocytes mainly through the glucose transporter 2 (GLUT2). Within the cells, free glucose is immediately phosphorylated on the sixth carbon by glucokinase (also named hexokinase IV), producing glucose-6 phosphate (G6P) and consuming one molecule of ATP. Contrary to the other hexokinases, glucokinase has relatively low affinity for glucose and is not inhibited by G6P [1]. Glucokinase expression is transcriptionally regulated by hormones [induced by insulin through the transcription factor SREBP1c (Sterol Response Element-Binding Protein 1c) and inhibited by glucagon] and metabolites of glucose and glucokinase activity is dependent on its binding to a specific inhibitor named glucokinase regulatory protein (GKRP) (see [2] for a review of glucokinase regulation). Other binding proteins such as 6-phosphofructo-2-kinase/fructose 2,6 biphosphatase (PFK2/FBP2) are also able to activate glucokinase by direct interaction with this enzyme [3].

The phosphorylation of glucose by glucokinase adds a charged phosphate group to this molecule. Consequently, G6P cannot cross the cell membrane, preventing the diffusion of free glucose out of the cells. Thanks to this phosphorylation step, glucokinase enables hepatocytes to trap glucose. During fasting periods, G6P is also produced after isomerization of glucose-1 phosphate during the breakdown of glycogen and by gluconeogenesis in the hepatocyte. It should be noted that a limited amount of free glucose can be directly released from glycogen through the action of the debranching enzyme α-1,6-glucosidase (AGL) and/or the lysosomal acid α-1,4 glucosidase (also known as acid maltase) [4].

**Figure 1:**
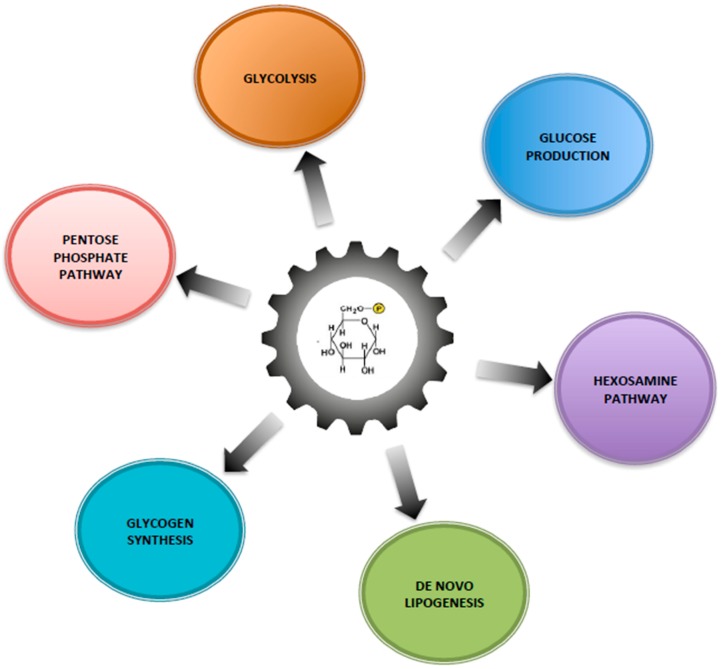
Glucose-6 phosphate, a central hub for liver carbohydrate metabolism. The increase of flux through G6P is responsible for increasing glycogen synthesis, glycolysis, pentose phosphate pathway (PPP), hexosamine pathway and de novo lipogenesis.

**Figure 2:**
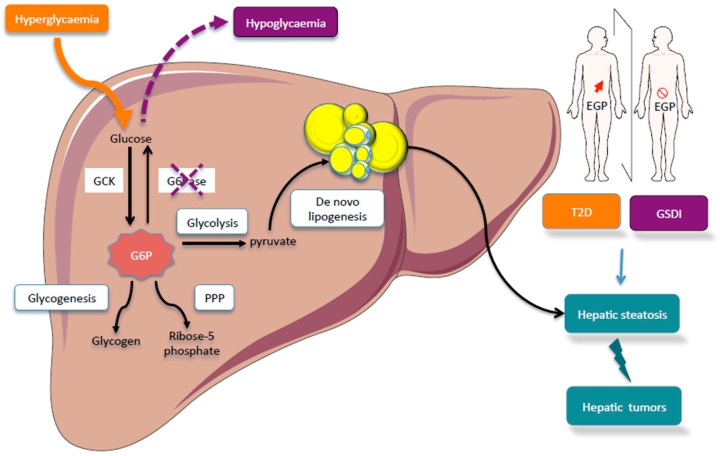
Comparison of hepatic glucose metabolism in glycogen storage disease type I (GSDI) or type 2 diabetes. Type 2 diabetes is characterized by an increase in endogenous glucose production (EGP) while GSDI is due to an absence of EGP. In GSDI, the absence of G6Pase activity is responsible for G6P accumulation in the hepatocyte. In diabetes, hyperglycemia is responsible for the increase flux through G6P. In both cases, this leads to a metabolic reprogramming characterized by the activation of glycolysis, PPP, and de novo lipogenesis. This metabolic reprogramming promotes hepatic steatosis in type 2 diabetes and GSDI, in which the risk of liver tumorigenesis is increased. Figures were drawn using Sevier Medical Art images.

Within the cells, G6P has many possible fates and therefore it represents a central hub for carbohydrate metabolism (Figure 1). After isomerization, it initiates major metabolic pathways, i.e., glycolysis, pentose phosphate pathway (PPP), glycogen synthesis, hexosamine pathway, and glucose production according to the nutritional or hormonal states. This review is focused on the key metabolic roles of G6P in cell signaling in the healthy or pathological liver. Here, we will highlight the metabolic reprogramming taking place in two metabolic diseases characterized by a dysfunction of glucose metabolism, namely type 2 diabetes and glycogen storage disease type I (GSDI). Interestingly, type 2 diabetes is an epidemic disease characterized by hyperglycemia, while GSDI is a rare genetic disease due to a loss of endogenous glucose production leading to severe hypoglycemia during short fasting. In type 2 diabetes, hyperglycemia is responsible for an increase in metabolic pathways downstream of G6P, while in GSDI the blockage of glucose production leads to the accumulation of G6P in the hepatocytes, which also increases all the metabolic pathways downstream of G6P (Figure 2). These two diseases are characterized by an accumulation of ectopic lipids in the liver, which leads to the development of hepatic steatosis and promotes hepatic tumorigenesis over time [5]. In this review, we will also consider the well-established role of the Carbohydrate-Responsive Element-Binding Protein (ChREBP) as the carbohydrate sensor that coordinates glucose and lipid metabolism in the liver according to nutritional states.

## 2. Metabolic Fate of Glucose-6 Phosphate in the Healthy Liver

In order to control cell metabolism and proliferation, G6P enters different metabolic pathways to provide energy and/or precursors for biomolecule synthesis needed to sustain these processes. First, glucose concentrations fluctuate between the fed state and fasting periods. The liver plays a crucial role in maintaining blood glucose levels by its capacity to produce glucose during fasting periods. Moreover, in the case of overnutrition, excessive G6P is converted into fatty acids via de novo lipogenesis in the liver. Secondly, during fasting periods, glucose should be preserved to supply precursors for maintaining biomass, especially for cell renewal. Ketone bodies then become a major energy source for most tissues. Thus, the liver plays a central role by coordinating the storage and synthesis of glucose and the redistribution of nutrients, through the G6P metabolism.

### 2.1. Glucose and Lipid Storage

After a meal, a large portion of the excess carbohydrates (approximately 30–40% of the glucose ingested) is stored as glycogen in the liver, inside the hepatocytes, and in muscles (glycogenesis). In healthy individuals, hepatic glycogen represents around 5% of the liver weight. Glycogen is a polymer of glucose residues linked by α-(1,4) and α-(1,6)-glycosidic bonds. To synthesize glycogen, G6P is isomerized into glucose-1 phosphate and then converted into UDP-glucose. For de novo glycogen synthesis, UDP-glucose molecules are attached to a protein known as glycogenin. Once a linear chain of 10–20 glucose moieties is formed, glycogen synthase extends the glycogen chain, forming α-1-4 glycosidic links, and a branching enzyme introduces a branch point. The branching enzyme transfers a glycosyl chain of 6 to 8 units to the glycogen thread forming an α-1-6 linkage [6]. G6P is a precursor for glycogen synthesis but it also plays a huge role in regulating the activities of glycogen synthase and glycogen phosphorylase. Indeed, G6P is an allosteric inhibitor of glycogen phosphorylase and an allosteric activator of glycogen synthase, thus favoring hepatic glycogen increase [7]. In addition, glycogen synthesis/degradation is tightly regulated by hormones and nutritional states, which has been extensively described (see [7] for a review). It is of note that the presence of high insulin level after a meal favors glycogen synthesis.

Importantly, the capacity to store glycogen in the liver is limited. In case of excessive feeding of carbohydrates or in pathological states such as GSDI, glycogen turnover allows to continually breakdown glycogen to limit glycogen accumulation [4,8]. Moreover, the excess of dietary glucose that cannot be stored as glycogen is converted into fat by de novo lipogenesis (see below) [9]. A deregulation of glycogen storage or metabolic dysfunctions leading to abnormal glycogen storage in the liver results in hepatic glycogen storage diseases, which are metabolic inherited diseases characterized by hypoglycemia. GSDI belongs to this group of hepatic diseases, representing about 30% of GSD cases [10].

In the liver, triglycerides can be packed into very low density lipoproteins (VLDL) and secreted into the circulation, stored as lipid droplets, or be metabolized by the β-oxidation pathway. Excessive G6P is converted into fatty acids via de novo lipogenesis using the acetyl-CoA generated from glycolysis-driven pyruvate and NADPH derived from PPP. After glucose load, lipogenesis is markedly increased at the expense of glycogen synthesis; conversely, low carbohydrate diets reduce de novo lipogenesis [9]. Interestingly, insulin secreted in response to elevated blood glucose levels and glucose can induce hepatic lipogenesis through the synergistically activation of SREBP-1c and ChREBP, respectively [11]. Thus, increased consumption of simple sugars leads to the ectopic accumulation of lipids in the liver and increases the risk of metabolic diseases such as obesity, type 2 diabetes and nonalcoholic fatty liver disease (NAFLD).

### 2.2. Maintenance of Glycaemia and Endogenous Glucose Production

The liver plays a key role in the maintenance of blood glucose, particularly during the beginning of fasting periods. Just after the intestinal glucose absorption from food is completed, hepatic G6P is mainly derived from glycogen breakdown, while gluconeogenesis becomes the major source of G6P after more prolonged fasting. Indeed, hepatic glycogen stores are depleted after a 12h-fasting in mice and an overnight fasting period in Humans [12,13]. Glycogenolysis requires the intervention of two different enzymes: glycogen phosphorylase that degrades the glycogen chain down to a chain length of 4 units into glucose-1 phosphate, and glycogen debranching enzyme (GDE) that first transfers 3 glucose units to the terminal end of another chain and then cleaves off the final glucose unit, releasing it as free glucose. Glucose-1-phosphate must further be converted by phosphoglucomutase into G6P to enter the metabolism mainstream. During a longer fast or starvation, the liver synthetizes glucose de novo mainly from lactate, alanine, and glycerol while glutamine is a predominant gluconeogenic substrate in the kidney and intestine [14,15]. Interestingly, the contribution of hepatic glucose production decreases during fasting [16]. This decrease is compensated by glucose production from the kidneys and intestine, which are especially capable of producing glucose thanks to gluconeogenesis and to participate in the maintenance of blood glucose when fasting is prolonged [17,18,19,20]. The significance of the renal and intestinal gluconeogenesis has been firmly demonstrated in mice that are incapable to produce glucose by the liver (Liver-specific *G6pc* knockout mice- L.G6pc^−/−^) [21]. Indeed, despite a drop in blood sugar levels in the post-prandial period, L.G6pc^−/−^ mice regulate their blood sugar similarly to control mice after several hours of fasting thanks to an induction of gluconeogenic genes in the kidney and the intestine [13,22].

To be released as glucose into the bloodstream, G6P has to be dephosphorylated into glucose by glucose-6 phosphatase (G6Pase), which is expressed only in the liver, kidneys, and intestine. G6P is first translocated into the endoplasmic reticulum by the G6Pase transporter subunit (G6PT) and subsequently hydrolyzed into free glucose and inorganic phosphate by the G6Pase catalytic subunit (G6PC). Glucose is finally released from the cytosol into the bloodstream through GLUT2. Thus, the liver, kidneys, and intestine play a central role in maintaining blood glucose levels at around 1 g/L (5 mM) since most mammals, including Humans, are incapable of tolerating hypoglycemia for more than a few minutes. Failure to activate these physiological pathways results in severe hypoglycemia that can be fatal, especially in GSDI or in diabetic patients treated with inappropriate doses of insulin.

### 2.3. Glucose-6 Phosphate: A Source of Energy and Carbon Skeletons

**Figure 3:**
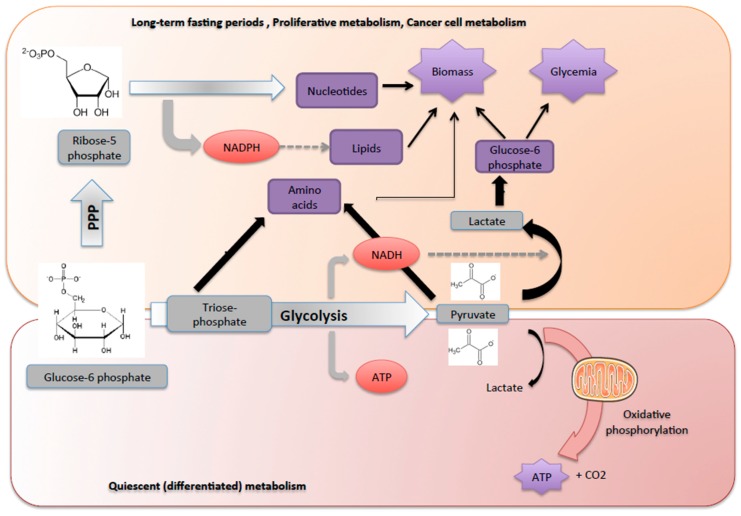
Glucose-6 phosphate: a source of energy and carbon skeletons. The G6P is metabolized either through the glycolytic pathway or PPP, which are tightly connected, depending on metabolic demands. Non-dividing normal differentiated cells mainly depend on mitochondrial oxidative phosphorylation of pyruvate, which is produced from glycolysis, to generate ATP. During cell proliferation or starvation periods, G6P is preferentially metabolized via PPP to maintain carbon homeostasis and produce biomass. In this case, glycolysis produces pyruvate and lactate as final metabolites and becomes inefficient in producing ATP. Indeed, G6P is preferentially metabolized via PPP to provide precursors for nucleotide and amino acid biosynthesis and to provide reducing molecules in the form of NADPH used in reductive biosynthesis reactions within cells (e.g., fatty acid synthesis). Lactate is also used by the hepatocyte to produce glucose and maintain glycaemia.

In feeding periods, glucose can be oxidized to CO_2_ through a series of metabolic pathways, namely glycolysis in the cytosol, followed by the tricarboxylic acid cycle and the respiratory chain in the mitochondria. The first step of glycolysis is the isomerization of G6P into fructose-6 phosphate to produce triose-phosphate, then resulting in the generation of 2 pyruvate molecules and a small amount of ATP (net gain of 2 ATP molecules). The oxidation of pyruvate then generates the bulk of ATP under aerobic conditions in quiescent differentiated cells (Figure 3).

**Figure 4:**
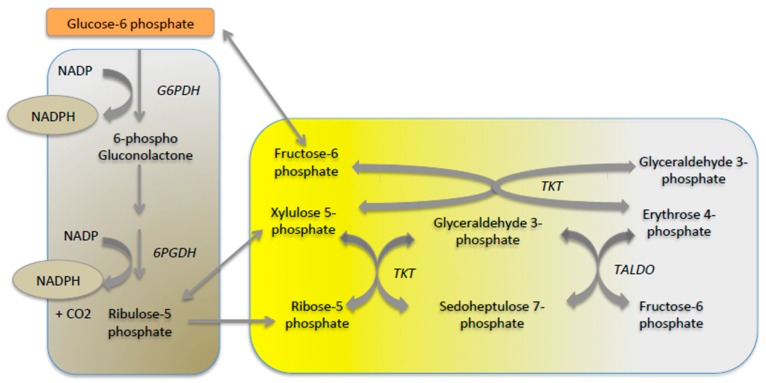
Scheme of the pentose phosphate pathway. The oxidative branch of PPP is highlighted in the brown part and the non-oxidative branch is represented in the yellow part of the figure. G6PDH: glucose-6 phosphate dehydrogenase; 6PGDH: 6-phosphogluconic dehydrogenase; TKT: Transketolase; TALDO: transaldolase.

While glucose is generally considered to be the main source of cell energy, it is above all a major provider of carbon skeletons for cell growth and survival [16]. Indeed, glucose oxidation to CO_2_ to produce energy should be avoided to permit to supply essential functions in some situations, in particular during long-term fasting or during cell proliferation. Glycolysis supplies 3 carbon-compounds, such as triose-phosphate, pyruvate and lactate that can be used to maintain cellular homeostasis and produce biomass (Figure 3). Hence, global glucose turnover decreases and glucose is used to supply PPP that provides the carbon skeletons needed for the synthesis of nucleotides, chromosomal duplication and cell proliferation (Figure 3). PPP is an important metabolic pathway known to provide reducing equivalents (NADPH) for anabolism and it plays a pivotal role in counteracting oxidative stress. Indeed, during oxidative stress, NADPH is needed for the generation of reduced glutathione. In the first step of PPP, G6P is oxidized into gluconolactone and carbon dioxide by glucose-6 phosphate dehydrogenase and 6-phosphogluconic dehydrogenase (oxidative branch). Ribulose-5 phosphate yielded is then isomerized to ribose-5 phosphate, which is the critical precursor for de novo ribonucleotide synthesis or epimerized into xylulose-5 phosphate. Additionally, a series of reversible reactions that recruit additional glycolytic intermediates, such as fructose-6 phosphate and glyceraldehyde-3-phosphate, can be converted into pentose phosphates and vice versa (non-oxidative branch). Transketolase (TKT) and transaldolase (TALDO) are the two major reversible enzymes that mediate the non-oxidative PPP and determine the diversion of metabolite flux in the PPP (Figure 4). Thus, in proliferative cells, TKT and TALDO divert fructose-6 phosphate and glyceraldehyde-3-phosphate from glycolysis to generate additional ribonucleotides. Interestingly, cancer cells can accelerate non-oxidative PPP by elevating the expression of these enzymes [23], while deficiency in TALDO can prevent HCC [24].

Thus, the ability to switch the glucose metabolism from a catabolic to an anabolic process is critical for cells to thrive, especially during long fasting periods. This capacity is also an advantage for cancer cells that can grow and multiply by using glucose as a carbon source to build proteins and nucleotides rather than as an energy source, thanks to the Warburg effect [25].

### 2.4. Hexosamine Pathway

When G6P is increased, the hexosamine pathway produces carbohydrate units for glycosylation of proteins and contributes to the synthesis of complex molecules such as glycolipids, proteoglycans and glycosylphosphatidylinositol anchors. First, G6P is converted into fructose-6 phosphate, which may either enter the hexosamine pathway in combination with glutamine to produce UDP-Nacetylglucosamine or it can follow the glycolytic pathway. The hexosamine pathway usually accounts for only 2–5% of total glucose metabolism. Interestingly, O-GlcNAcylation of different key transcription factors involved in energy metabolism, including ChREBP and the nuclear receptor Farnesoid X receptor (FXR), requires the hexosamine pathway [26].

## 3. ChREBP: A Glucose Sensor

In the hepatocyte, the transcriptional effects of glucose on gene expression are mediated by the transcription factor ChREBP, which in interaction with Max-like protein (Mlx) binds conserved consensus sequences (Carbohydrate Response Element, ChoRE). Indeed, in response to increased glucose concentration, ChREBP is translocated to the nucleus and it activates several genes involved in glucose and lipid metabolism [such as liver-Pyruvate kinase (L-PK), Fatty Acid Synthase (FAS) acetyl-CoA carboxylase (ACC), and stearoyl-CoA desaturase (SCD1)], but also genes involved in insulin signaling [27,28,29]. Recently, ChREBP was pointed out as a potential regulator of VLDL secretion in the liver [30]. Thus, it is assumed that ChREBP has important roles in the development of liver diseases including NAFLD [31]. In consequence, inactivation of ChREBP or liver-specific inhibition of ChREBP led to a decrease in glycolytic and lipogenic gene expression and a decrease in hepatic steatosis in mice [29,32,33]. On the contrary, the overexpression of ChREBP led to the development of hepatic steatosis without concomitant insulin resistance [27]. More recently, a key regulatory role for ChREBP in hepatic tumorigenesis was also suggested, since ChREBP expression was found to be increased in non-tumorous surrounding tissue in liver samples and further increased in HCC in Humans [34]. In addition, a recent study reported the importance of ChREBP in HCC [35]. The genetic deletion of ChREBP in mice impaired hepatocarcinogenesis driven by protein kinase B/Akt overexpression [36]. Furthermore, in vitro studies of ChREBP silencing in hepatoma cells resulted in a metabolic switch from aerobic glycolysis to mitochondrial oxidative phosphorylation, concomitantly with a reduction of cell proliferation [37]. In GSDI, the overexpression of ChREBP has been linked to glucose and lipid metabolism reprogramming [38,39]. In this context, enhanced ChREBP could partly account for increased proliferation of hepatocytes by favoring cancer cell-like metabolism. Further investigation is required to unravel the exact role of ChREBP in hepatocarcinogenesis in the context of NAFLD.

Two isoforms of ChREBP have been recently described originating from an alternative first exon promoter - ChREBP α and β [40]. The presence of a ChoRE sequence in the exon promoter 1β suggests that ChREBPα directly regulates the expression of ChREBPβ, considered as a constitutively active isoform (due to the loss of a regulatory inhibitory domain). Consequently, the response to glucose under hyperglycemic conditions could be exacerbated. The regulation of ChREBP activity by glucose is complex and the relative role of xylulose-5 phosphate, G6P or other glucose metabolites on the triggering of ChREBP activation is still discussed [41]. It was shown that xylulose-5 phosphate activates PP2A promoting dephosphorylation of ChREBP and its nuclear translocation and activation [42]. However, G6P seems to have a central role for the increase in ChREBP activity, especially by favoring ChREBP translocation to the nucleus and transactivation [28]. The key role of G6P was supported by the identification of a putative G6P recognition motif in the transactivation domain, called glucose-activation conserved element (GRACE), suggesting the possibility of an allosteric regulation of ChREBP by G6P [43]. High glucose also stimulates ChREBP activity and affinity to ChoRE sequences through acetylation and/or O-GlcNacetylation [44,45]. Finally, during fasting periods, phosphorylation of ChREBP by AMPK, in response to glucagon or to an increase in cellular AMP, is responsible for its cytoplasmic retention and/or for its decreased binding to target promoters [46,47].

In conclusion, ChREBP is a carbohydrate-signaling transcription factor, which masters, in the liver, the storage of lipids in feeding response. Recent studies have also supported the importance of ChREBP in the regulation of fructose metabolism [48,49].

## 4. Imbalance of Glucose-6 Phosphate Metabolism Leads to Metabolic Diseases and Promotes Hepatocarcinogenesis

In this review, we have chosen to illustrate two different pathological states characterized by impaired glucose metabolism. Firstly, type 2 diabetes is a chronic disease with the status of a global pandemic that is closely linked to overnutrition and obesity [50,51]. The main hallmark of diabetes is hyperglycemia due to insulin resistance and an overproduction of glucose by the body [52]. Hyperglycemia results in non-enzymatic glycosylation (glycation) and thus loss of function of proteins, glucose-induced oxidative damage and other adverse effects such as macrovascular and microvascular complications [53]. The second metabolic disease characterized by carbohydrate metabolism disruption is GSDI. This is a rare genetic disease (1 birth over 100,000) due to mutations in *G6PC* (that cause GSDIa) or *G6PT* (that cause GSDIb) leading to a loss of G6Pase activity and endogenous glucose production. In consequence, patients develop severe hypoglycemia during short fasting periods [54]. Thus, although type 2 diabetes and GSDI appear to be opposite diseases in terms of glucose production and insulin sensitivity, the liver is chronically exposed to either hyperglycemia or G6P accumulation, respectively, leading the same metabolic consequences, in particular hepatic steatosis (Figure 2). Comparing type 2 diabetes and GSDI will allow us to highlight metabolic perturbations that promote tumour development in relation to the ectopic accumulation of lipids in the liver.

In both type 2 diabetes and GSDI, liver metabolism is characterized by an increased metabolic flux downstream of G6P (Figure 2). Even if glucose uptake is impaired in obese and/or diabetic mice or patients, high blood glucose levels are responsible for the activation of all G6P-dependent pathways previously described [7]. Subsequently, one major metabolic consequence is an increase in triglyceride synthesis by the liver leading to hepatic steatosis [55,56]. Indeed, up to 70% of diabetic subjects may present NAFLD [56,57] and all GSDI develop a NAFLD-like pathology [54]. In insulin-resistant states, hyperglycemia and hyperinsulinemia are in part responsible for enhancing de novo lipogenesis through the activation of both ChREBP and SREBP1c. Interestingly, both transcription factors are also induced in the context of a high carbohydrate feeding independently of insulin signaling [58]. As previously mentioned, it has been shown that the global or liver-specific inhibition of ChREBP protected mice against carbohydrate-induced hepatic steatosis [29,33]. However, the effects of ChREBP inhibition on hepatic insulin sensitivity are still controversial. In GSDI, the absence of G6Pase activity leads to the accumulation of G6P in the liver and consequently the accumulation of glycogen and lipids, responsible for hepatomegaly and hepatic steatosis. Contrarily to diabetes, lipid synthesis is activated by ChREBP but independently of liver X receptor (LXR) and SREBP-1c [59]. The lack of SREBP-1c activation is probably due to a low intensity of insulin signaling in GSDI [37]. De novo lipogenesis is not the only process contributing to fatty liver. Indeed, the accumulation of lipid is also caused by an unbalanced diet, elevated non-esterified fatty acid due to a decreased inhibition of adipose tissue lipolysis, and reduced hepatic VLDL export [60,61]. All these disturbances contribute to hypertriglyceridemia and hypercholesteridemia observed in diabetes and GSDI. In conclusion, the liver metabolism of diabetes and GSDI is very similar, albeit exacerbated in GSDI, with G6P being at the metabolic crossroad as a main responsible for metabolic reprogramming [39,62].

Interestingly, both diabetes and GSDI patients are prone to the development of hepatic tumors. In diabetes, NAFLD can progress to liver fibrosis associated with inflammation i.e., non-alcoholic steatohepatitis NASH, cirrhosis and finally to the development of HCC. However, an important fraction of obese/diabetic patients develop HCC in the absence of liver cirrhosis [63,64]. Interestingly, GSDI subjects develop simple hepatic steatosis, which was long considered as a benign reversible condition. Nonetheless, lipid accumulation in the liver is a fertile ground for the development of hepatic tumors and most of patients with GSDI develop hepatocellular adenomas (HCA) that can later progress into HCC [54]. Despite the important accumulation of glycogen and lipids, GSDI patients present only low-grade hepatic inflammation and no hepatic injuries (namely normal hepatic transaminase levels and absence of liver failure). It is noteworthy that in obese/diabetic patients a part of HCA arises at the state of NAFLD characterized by a low-grade inflammation and may progress to HCC [65]. The comparison of HCC occurrence in NAFLD and GSDI livers argues for a dominant role of metabolic reprogramming in the molecular induction of tumor development.

Interestingly, tumor cells are metabolically reprogrammed to fuel cell proliferation, mostly by increasing glucose uptake and flux through aerobic glycolysis (Warburg effect) and anabolic pathways (PPP and de novo lipogenesis). The Warburg effect is characterized by high rates of glycolysis and lactic acid fermentation that occur in the cytosol regardless of the oxygen level. This provides essential bioenergetic substrates for cell growth and replication, i.e., components needed for cellular membrane biogenesis and amino acids and nucleotide synthesis for cell division. Recently, we showed that GSDI hepatocytes exhibit the main characteristics of cancer cell metabolism, with a Warburg-like metabolic reprogramming that predisposes GSDIa livers to tumor development [15]. Indeed, we observed a hyperactivation of the glycolysis pathway notably characterized by an overexpression of the M2 isoform of pyruvate kinase in the tumors and an increase in lactate production. Moreover, OXPHOS analyses revealed a decrease in mitochondrial respiration with a reduction of pyruvate oxidation [39].

A rational therapeutic approach for the treatment of NAFLD is to increase hepatic energy expenditure and thereby increase hepatic fat oxidation. Recently, we showed that the use of PPAR-α agonists, in particular fenofibrate, prevented NAFLD and hepatic injuries in GSDI, as previously described in diabetes [66,67,68]. Interestingly, the activation of β-oxidation by fenofibrate promoted the utilization of G6P through lipid metabolism, avoiding the accumulation of glycogen [66]. In diabetes, thyroid hormone receptor-β agonist combined with glucagon treatment, or glucacon like peptide 1 agonist-gastric inhibitory peptide-glucagon tri-conjugate [69], or liver targeted mitochondrial protonophores [70,71] were shown to reverse NAFLD in preclinical studies. Interestingly, glucose-lowering medications such as metformin also reduce the risk of HCC in diabetes, suggesting that better control of hepatic glucose metabolism should permit prevention of carcinogenesis.

To conclude, the activation of G6P-mediated metabolism is a hallmark of both GSDI and diabetes that causes hepatic steatosis and may promote cell proliferation and liver cancer. Thus, an optimal metabolic control, thanks to a strict diet with a reduced consumption of simple carbohydrates, should prevent tumor occurrence in GSDI [54]. In diabetes, better control of hyperglycemia should also permit better control of glucose/G6P metabolism and its possible consequences in hepatocytes. Thus, comparing these two metabolic diseases is a useful approach to better understand the key role of G6P in the liver both in health and pathological conditions.
